# Comment on Gassenmaier et al. Accelerated T2-Weighted TSE Imaging of the Prostate Using Deep Learning Image Reconstruction: A Prospective Comparison with Standard T2-Weighted TSE Imaging. *Cancers* 2021, *13*, 3593

**DOI:** 10.3390/cancers15020370

**Published:** 2023-01-06

**Authors:** Francesco Pucciarelli, Andrea Laghi, Damiano Caruso

**Affiliations:** Radiology Unit, Department of Medical Surgical Sciences and Translational Medicine, Sapienza University of Rome-Sant’Andrea University Hospital, Via di Grottarossa, 1035-1039, 00189 Rome, Italy

Magnetic resonance imaging (MRI) plays a central role in oncology without using ionizing radiation or radioactive markers. MRI is characterized by various features that render it superior to computed tomography (CT) scanning: greater soft-tissue contrast and improved tissue characterization based on the signal behavior of different pulse sequences and relaxation parameters [1]. 

Prostate cancer represents the highest cause of cancer mortality in Western countries, especially in the male population aged between 45 and 60 years [2]. Risk factors include family risk, ethnicity, age, obesity, and other environmental factors, and it can be classified as androgen-sensitive or androgen-insensitive (this is an indicator of testosterone stimulation). Treatment options include active surveillance, chemotherapy, radiation therapy, hormonal therapy, surgery, and cryotherapy [3].

MRI has represented the main modality for the noninvasive assessment of the prostate gland and surrounding structures since the 1980s. Initially, prostate MRI was based solely on morphologic assessment using T1-weighted (T1W) and T2-weighted (T2W) pulse sequences, and its role was mainly in locoregional staging in patients with biopsy-proven cancer. Advances in technology (both in software and hardware) have led to the development of multiparametric MRI (mpMRI), which combines anatomic T2W, diffusion-weighted imaging (DWI) and its derivative apparent-diffusion coefficient (ADC) maps, dynamic contrast-enhanced (DCE) MRI, and sometimes other techniques such as in vivo MR proton spectroscopy. Consequently, clinical applications of prostate MRI have expanded to evaluate not only locoregional staging, but also lesion detection, localization, characterization, risk stratification, surveillance, the assessment of suspected recurrence, and image guidance for biopsy, surgery, focal therapy, and radiation therapy. The prostate imaging reporting and data system (PI-RADS v2.1) is a scoring system created to improve detection, localization, characterization, risk stratification, and outcomes in patients with suspected prostate cancer. The specific aims are to establish minimum standard technical parameters for prostate mpMRI; simplify and standardize the terminology and content of radiology reports; enable the use of MRI data for targeted biopsy; develop assessment categories that summarize levels of suspicion or risk and can be used to select patients for biopsies and management (e.g., observation strategy vs. immediate intervention); and allow data collection and outcome monitoring. Furthermore, it is important to train radiologists in prostate MRI reporting and reduce variability in imaging interpretations for better interdisciplinary communications with referring clinicians [4].

In recent years, artificial intelligence (AI)—in particular, deep learning (DL)—has been gaining ground in many areas of imaging, such as image classification, segmentation, denoising, super-resolution, and the synthesis/transformation of the image [5]. DL algorithms allow the reconstruction of images with higher SNR, improved spatial resolution, and reduced truncation artifacts, after such algorithms have been trained on a high volume of high-quality images considered as ground truth. This results in images with high diagnostic quality and reduced acquisition times compared with standard protocols [6,7]. Many DL algorithms focus on accelerated image acquisition, as demonstrated by many authors in different anatomical districts [8,9].

**The Study by Gassenmaier et al. Published in *Cancers***

In their study, Gassenmaier et al. [10] aimed to investigate the impact of the DL reconstruction algorithm in accelerated T2 TSE imaging of the prostate in three orthogonal planes on image quality, lesion conspicuity, and diagnostic confidence, compared with standard T2 TSE imaging. The authors demonstrated how the application of a DL algorithm applied to the acquisition of the protocol of the mpMRI on 3T scanner allows T2-weighted images to be obtained on three orthogonal planes in a significantly reduced amount of time compared with conventional T2 sequences. In addition to acquiring images in a significantly shorter amount of time, they have a higher—or in any case, no lower—quality than standard images. Furthermore, no significant differences between the PI-RADS score and the T2 score were observed between sequences.

This was a prospective study in which 60 patients were enrolled, starting from an initial population of 134. All patients underwent mpMRI examination for the study of the prostate and were acquired on three different 3T scanners. The acquisition protocol consisted of the following sequences: T2w TSE imaging in three planes; DWI with three different acquired b-values in axial plane (50 s/mm^2^, 500 s/mm^2^, and 1000 s/mm^2^) as well as one calculated b-value of 2000 s/mm^2^ and ADC mapping; and for the evaluation of possible bone lesions and lymph node status, a T1w precontrast TSE image with a larger field of view was obtained. Furthermore, after the application of contrast media (0.1 mmol/kg body weight gadobutrol; Gadovist, Bayer Healthcare) using a flow rate of 1.5 mL/s followed by a saline flush of 20 mL, dynamic contrast-enhanced gradient echo imaging was acquired in the axial direction followed by an additional post-contrast gradient-echo axial sequence. After the completion of standard T2w TSE imaging (T2S), the novel T2w TSE image with deep learning image reconstruction (T2DLR) was acquired using a prototype sequence.

Overall, the acquisition time for T2S resulted in 10:21 min versus 3:50 min for T2DLR.

In addition to comparing the acquisition times, the authors also demonstrated the results in terms of the qualitative analysis of the images. The analysis of the images was performed by two radiologists with a different number of years of experience, who evaluated the T2 score and PI-RADS score according to PI-RADS v.2.1, location, and lesion size. Furthermore, noise levels, lesion conspicuity, the magnitude of artifacts, the diagnostic confidence of the readers, and overall image quality were evaluated with a Likert scale ranging from 1 to 4. 

The results demonstrate that the application of this DL algorithm on T2-w sequences for the mpMRI of the prostate was able to reduce the acquisition time by approximately 60% and showed a superior image quality compared with the images acquired with the standard protocol. No significant differences were demonstrated regarding the PI-RADS score and T2 score between the two sequences.

The interesting results of this study should be considered in light of some limitations, as underlined by the authors. In particular, this DL algorithm was applied only on T2-weighted sequences and not on the remaining sequences; furthermore, there was no correlation with the pathological data obtained by biopsy. 

Therefore, these can be considered promising preliminary data regarding the application of DL algorithms to the study of the mpMRI of the prostate. In the future, it could be very interesting to obtain results from the application of this algorithm on other sequences of the standard mpMRI protocol, in terms of both acquisition time and image quality. Furthermore, as well as results from qualitative data, it could also be very interesting to obtain quantitative results (e.g., radiomic analysis).

These results are in agreement with the results obtained by other authors regarding the application of DL algorithms in the study of other anatomical districts. Thus, these algorithms may be of great importance for establishing ultrafast screening protocols in prostate MRI, and could be especially useful in claustrophobic or uncooperative patients. 
